# Principles for Just Prioritization of Expensive Biological Therapies in the Danish Healthcare System

**DOI:** 10.1007/s11673-023-10283-2

**Published:** 2023-09-21

**Authors:** Tara Bladt, Thomas Vorup-Jensen, Mette Ebbesen

**Affiliations:** 1 Danish College of Pharmacy Technicians, Hillerød, Denmark; 2 Department of Biomedicine, Aarhus University, Aarhus, Denmark; 3 Techno-Anthropology & Participation, Department of Planning, Aalborg University, Rendsburggade 14, DK- 9000 Aalborg, Denmark

**Keywords:** Principles, Prioritization, Justice, Expensive therapies, Healthcare system

## Abstract

The Danish healthcare system must meet the need for easy and equal access to healthcare for every citizen. However, investigations have shown unfair prioritization of cancer patients and unfair prioritization of resources for expensive medicines over care. What is needed are principles for proper prioritization. This article investigates whether American ethicists Tom Beauchamp and James Childress’s principle of justice may be helpful as a conceptual framework for reflections on prioritization of expensive biological therapies in the Danish healthcare system. We present an empirical study exploring the principles for prioritizing new expensive biological therapies. This study includes qualitative interviews with key Danish stakeholders experienced in antibody therapy and prioritizing resources for expensive medicines. Beauchamp and Childress’s model only covers government-funded primary and acute healthcare. Based on the interviews, this study indicates that to be helpful in a Danish context this model should include equal access for citizens to government-funded primary and acute healthcare, costly medicine, and other scarce treatments. We conclude that slightly modified, Beauchamp and Childress’s principle of justice might be useful as a conceptual framework for reflections on the prioritization of expensive biological therapies in the Danish healthcare system.

## Introduction

The Danish Health Act states that the healthcare system aims to promote the general health of the Danish population while preventing and treating illness, pain, and disability in the individual patient. Moreover, the Danish healthcare system must meet the need for easy and equal access to healthcare for every citizen (Danish Health Act 2019). However, the Danish Council on Ethics (DCE) published a report in 2018 stating that the Danish healthcare system does not adhere to the principle of equal access for every citizen. The report criticized the Danish healthcare system for 1) unfair prioritization of resources for expensive medicines rather than for care and 2) unfair prioritization of specific diseases, for example, patients with cancer rather than patients suffering from severe chronic obstructive pulmonary disease or schizophrenia (Danish Council on Ethics 2018, 3–17).

The DCE stresses that principles for prioritization are needed in the Danish healthcare system (Danish Council on Ethics 2018, 3). This is in line with the position of the American ethicists Tom Beauchamp and James Childress, who note that some countries lack an inclusive and coherent healthcare financing and distribution system and experience a continued increase in expenditures and in the number of citizens who are not protected. According to Beauchamp and Childress, these countries must improve efficiency, fairness, and equality since these values are necessary for modelling a just healthcare system. Both utilitarian perspectives of efficiency and egalitarian perspectives of universal access must be included in reflections on prioritization in healthcare, with trade-offs between equality and efficiency (Beauchamp and Childress 2019, 313).

Starting with the DCE statement that principles for prioritization are needed in the Danish healthcare system (Danish Council on Ethics 2018, 3), this article explores whether Beauchamp and Childress’s (2019) principle of justice may be helpful as a conceptual framework for reflections on the prioritization of expensive biological therapies in the Danish healthcare system. First, we identify ethically important features of antibody therapies. Next, we present the Danish Medicines Council (DMC) and its task of evaluating whether new medicines should be introduced as standard treatments in Danish hospitals. We then introduce the Beauchamp and Childress (2019) ethical principle of justice and present a qualitative study in which semi-structured interviews were conducted with critical stakeholders as informants experienced in antibody therapy or prioritizing resources for expensive medicines in Denmark (Bladt et al. 2020). The validity and relevance of the study are considered, and the results are discussed in the light of Beauchamp and Childress’s theory (2019).

## Background

### A Brief Outline of Antibody Therapies and Their Role in Modern Healthcare

Antibodies are proteins made by immune cells in response to foreign or “non-self” molecules that may reflect the presence of microbial infection. Antibodies that bind parts of the microbial threat serve to eliminate the danger itself or neutralize certain functions of the microbial molecules, for example, those permitting the microbes to stick to human tissues or the impact of microbial toxins (Murphy, Weaver, and Berg 2022). The production of recombinant antibodies that react with targets of choice, including molecules of our own body, has revolutionized therapies for cancer and autoinflammatory diseases. Over the last thirty years, the use of these therapies has increased significantly, both in the number of indications targeted and the wider use of specific antibodies for more diseases. Most of these formulations are costly, with a complex development in price (Lu et al. 2020). Interfering with biological processes in a controlled fashion by injection of antibodies induces a strong immune system response to malignant tissue and suppresses autoimmune disease. We refer to these medicines as “biological therapy,” because the basis of the therapy is a molecule of biological origin, unlike many other treatments that are based on molecules made by chemical synthesis. As mentioned above, they have proven beneficial in reducing inflammation associated with a diverse range of diseases, including rheumatoid arthritis (Furst et al. 1999) and multiple sclerosis (MS) (Mariottini, Muraro, and Lünemann 2022) and more recently in increasing the immune response to several cancer types (Sharma and Allison 2020).

Before the introduction of biological therapies, medical treatments for these diseases and several others were highly unsatisfactory. For instance, in the case of rheumatoid arthritis, extensive damage to the joints, previously a common long-term outcome, is far less frequent nowadays among patients with access to biological therapy. For MS patients, the onset of severe disability is significantly delayed. Neither therapy offers a cure for the disease, so the treatment is lifelong. Biological therapy has rarely challenged conventional medical ethics. Gains in health benefits outweigh concerns over side effects. However, after three decades of using biological therapies, a complex impact on the healthcare system is emerging in the form of a dilemma. On the one hand, in diseases with no cure such as MS, symptoms can now be alleviated to an extent that allows a significantly improved quality of life. On the other hand, the high price of such therapy imposes a significant economic burden on healthcare systems but one that cannot easily be rejected because the therapies are mostly successful (Parker-Lue, Santoro, and Koski 2015).

Hartung discusses this topic, noting that the soaring prices in MS therapy are attributable to market dysfunction from a lack of price transparency, monopolistic conditions, uncertain clinical evidence, and a broken market with payer and supply-chain issues (Hartung 2017). Hartung is undoubtedly right about the pricing of many biological therapies. While strict comparison between the cost development of different types of medication is highly complex, there is little doubt that biological therapies are more expensive than most of the drugs they replace. This assessment rests on the high overall risk-taking of making effective drugs that deal with multifactorial diseases and the high manufacturing costs of the requirements of biological systems, which essentially limit production to highly specialized facilities and raise the entry-level costs for bringing competing drugs to the market. Other factors may include physician ignorance of biosimilar availability (Troein et al. 2021) or their general unwillingness to use these alternatives based on personal experience of differences in, for instance, side effects compared to the original product (Bladt et al. 2020).

Biological therapies are therefore likely to remain a significant economic burden on healthcare systems, which gives rise to several questions tightly linked with ethical considerations relevant to healthcare systems. As we have discussed elsewhere (Bladt et al. 2020), if one particular therapy comes at a high economic cost, it may exclude the possibility of offering other treatments in a healthcare system with finite budgets. Moreover, the rapid increase in available treatments, combined with the fact that the disease indications in question now include relatively common morbidities, for example, rheumatoid arthritis, means there are many more patients to treat. Complex choices of what therapies to use or relinquish seem inescapable, including weighing the emotional inclination of wanting to help diseased individuals with their ailments against the per-definition utilitarian basis of drug efficacy and healthcare economics.

### The Danish Medicines Council and Prioritization

In Denmark, prescription medicine is evaluated by the DMC, which recommends 1) whether new treatments should be available as standard treatments and 2) the medicines healthcare professionals should use in Danish hospitals. The DMC evaluates the effect of treatments, comparing it with the medicine’s side effects and price. This work aims to ensure both up-to-date treatments for patients and the utilitarian objective of maximum health for the money spent. Similar systems for prioritizing hospital medicines are found in Sweden (Dental and Pharmaceutical Benefits Agency 2022) and Norway (Norwegian Ministry of Health and Care Services 2017).

The DMC consists of three units: the Council, the Expert Committees, and the Secretariat. The Council makes the final decisions. Its members are physicians in leading positions across the country, a health economist, clinical pharmacologists, a hospital apothecary, and two patient representatives. The Expert Committees focus on specific disease areas, and their members include physicians, pharmacologists, and patient representatives. The Secretariat serves the Council and the Expert Committees (Danish Medicines Council 2022).

When the DMC was established in 2017, the Danish parliament adopted seven principles for prioritizing expensive medicine in Danish hospitals. The first principle is “professional competency,” meaning that the decisions made by the Council must take professional expertise and knowledge into consideration and that the evaluations are systemically made (Danish Ministry of Health 2016). The remaining six principles are 2) independence, 3) geographical equality, 4) openness, 5) fast use of new and effective medicine, 6) more health for money, and 7) access to treatment. The Danish Regions supplemented these principles with two important factors to consider in medicine evaluations: 1) severity and 2) caution (Danish Council on Ethics 2018, 12) (text box 1). The Council describes how treatment recommendations are made and the factors that are measured, for example, when two treatments are compared.

Text box 1. Principles for prioritization of medicine in Danish hospitals (Danish Ministry of Health 2016).

**Table:** 

**Principles and important factors for prioritization of medicine in Danish hospitals**
**Principles:**
*1) Professional competency* Thorough and systematic evaluation of medicine.
*2) Independence* Objective criteria for evaluation not influenced by political agendas.
*3) Geographic equality* Equal use of medicine across the country.
*4) Openness* Transparent evaluation of medicine.
*5) Fast use of new and effective medicine* Denmark as a leading county for the use of new medicine with a documented effect.
*6) More health for the money* Reasonable ratio between effect and price of medicine.
*7) Access to treatment* Equal access to medicine for minor and major patient groups.
**Important factors:**
*1) Severity*
*2) Caution*

When biosimilars are introduced, the manufacturer must apply to the European Medicines Agency (EMA) for marketing authorization and document that there is no difference in effect, safety, and quality from the original drug. The documentation must show that variations in the molecular structure between the biosimilar and the original drug do not influence effect and safety. The Council then decides which patient groups will change over to the biosimilar drug (Danish Medicines Council 2017).

In January 2021, the Council started using quality-adjusted life-years (QALYs) in their analyses along with the seven principles and the two important factors for prioritization. This was intended to ease the comparison of treatments across disease areas and make more transparent decisions (Danish Medicines Council 2022) and follows the DCE recommendation that the Council should apply QALYs supplemented with other perspectives to be able to compare different types of diseases (Danish Council on Ethics 2018, 16).

## Theory

### Justice in National Healthcare Systems

Beauchamp and Childress recommend a just organization of a healthcare system with two tiers. Tier 1 is financed by the government covering universal access for primary and acute healthcare (a decent minimum^1^) for every citizen, while Tier 2 is self-financed, covering other health needs. This model incorporates egalitarian ideals of equal access and fair opportunity (Tier 1) and the utilitarian ideals of promoting social utility based on cost-effective analyses (Tier 2) (Beauchamp and Childress 2019, 293). The two-tiered model safeguards everybody by ensuring universal access to basic health needs, thereby admitting “that society’s obligations are demanding, but not limitless” (Beauchamp and Childress 2019, 293).

The Danish healthcare system is organized in line with Tier 1 in this model. Importantly, however, the Danish healthcare system does not only cover primary and acute healthcare. Nevertheless, citizens have to co-finance treatments at the dentist and medicine bought at the local pharmacy (biological therapies are in most cases administered by injection at hospital departments, with costs covered by the department). In Tier 2, citizens have access to private hospitals at their personal expense. The DCE specifically refers to Beauchamp and Childress’s (2019) theory regarding the fairness of a decent minimum of healthcare for every citizen (Danish Council on Ethics 2018, 13).

Beauchamp and Childress justify the universal right to a decent minimum of healthcare funded by the government by reference to the fair-opportunity rule, which implies that

the justice of social institutions should be assessed by their tendency to counteract lack of opportunity caused by unpredictable misfortune over which the person has no meaningful control. The need for health care is greater among the seriously diseased and injured, because the costs of health care for them can be uncontrollable and overwhelming, increasingly so as their health status worsens. Insofar as injuries, diseases, or disabilities create profound disadvantages and reduce agents’ capacity to function properly, justice as the provision of a fair opportunity requires that we use societal health care resources to counter these effects and give persons a fair chance to develop, maintain, restore, and use their capacities. (Beauchamp and Childress 2019, 292)

The two-tiered system of healthcare allows the utilitarian approach to base allocation decisions on cost-effectiveness and cost-benefit analyses with the goal of “the greatest health benefits for the money expended” (Beauchamp and Childress 2019, 303). However, according to Beauchamp and Childress, cost-effectiveness and cost-benefit analyses^2^ fail to attend adequately to important concerns of justice “including the needs of people with disabilities and the needs of the worst off in terms of the severity of their current illness and their health over a lifetime” (Beauchamp and Childress 2019, 255). The use of cost-effectiveness and cost-benefit analyses should therefore be constrained by the right to a decent minimum of healthcare and the fair-opportunity rule (Beauchamp and Childress 2019, 256, 293). Beauchamp and Childress write:

In our judgment, data gained from CBA^3^ and other analytic techniques can be made relevant to the formulation and assessment of public policies and can provide valuable information and insights if appropriate qualifications and limits are articulated, but they provide only one set of indicators of appropriate social beneficence. (Beauchamp and Childress 2019, 253)

So, Beauchamp and Childress specifically acknowledge that cost-effectiveness and cost-benefit analyses provide important information but argue that these analyses should be supplemented with important reflections on justice, fairness, and equity (Beauchamp and Childress 2019, 256). In line with this in the Danish context, the Council’s principles and important factors for prioritization explicitly state that the Council must apply perspectives of equal access, severity, and caution when evaluating new treatments (text box 1). However, the DCE points out that it is not transparent how or whether this is actually done. For instance, it is unclear whether patients suffering from rare diseases (minor patient groups),^4^ for whom medicine is often expensive and not well-documented, are guaranteed equal access compared to patients suffering from common diseases (major patient groups).^5^ Furthermore, the DCE points out that the Danish Health Act does not explicitly state the specific amount of basic medical examination and treatment to which every citizen is entitled (Danish Council on Ethics 2018, 13–15).

According to Beauchamp and Childress, the principle of justice is only one principle in a cluster of universal ethical principles appropriate for biomedical ethics. This cluster of principles also includes beneficence, nonmaleficence, and respect for autonomy. These principles are equally important and prima facie binding, meaning that each principle “must be fulfilled unless it conflicts with an equal or stronger obligation” (Beauchamp and Childress, 2019, 15). When applied, the principles are specified and balanced (Beauchamp and Childress 2019, 15).

### Ethical Theory of Principles: Structuring the Data Analysis

In Bladt et al. (2020), we presented an empirical study examining ethical issues of prioritizing new and expensive biological therapies in the Danish healthcare system. Beauchamp and Childress’s four principles of beneficence, nonmaleficence, justice, and respect for autonomy were used to structure the data analysis (Bladt et al. 2020). This article focuses exclusively on justice, specifically the principles for prioritizing new expensive biological therapies in the Danish healthcare system. It is based on the same interviews with respondents recorded by Bladt et al. (2020), but all the interviews were re-analysed and re-interpreted using Beauchamp and Childress’s ethical principle of justice as an open framework to structure the data analysis.

From its Danish context, one might wonder why this study was not structured by the European ethical principles presented by Rendtorff and Kemp (2000) on autonomy, dignity, integrity, and vulnerability (Druedahl, Lebret, and Minssen 2020). Specifically, Druedahl, Lebret, and Minssen argue that choosing Beauchamp and Childress’s theory (2019) overlooks the principle of solidarity, even though this principle is important in a Danish context (Druedahl, Lebret, and Minssen 2020). Nevertheless, we selected Beauchamp and Childress’s (2019) theory because it is one of the most influential theories in biomedical ethics and has been used worldwide for ethical assessments for more than four decades. Beauchamp and Childress’s theory is heavily debated in the literature. We have argued that the criticism is not substantial and that the theory is well justified both normatively and empirically (Ebbesen 2011). For instance, at least two qualitative studies indicate that Beauchamp and Childress’s theory of principles is applicable in Danish biomedical practice (Bladt et al. 2020; Ebbesen and Pedersen 2007a). Moreover, the DCE specifically refers to this theory in their report on just prioritization in the Danish healthcare system (Danish Council on Ethics 2018, 13). While Rendtorff and Kemp’s theory may accommodate certain national patterns of ethical priorities, it also has philosophical shortcomings, as explained elsewhere (Beauchamp 2019; Ebbesen 2010).

Indeed, Rendtorff and Kemp propose their theory as a European alternative to Beauchamp and Childress’s theory that preserves “the fragile and finite, bodily incarnate human person” (Rendtorff and Kemp 2000, 18–19, 314; Ebbesen 2010, 96, 103). Rendtorff and Kemp charge Beauchamp and Childress’s theory with having a minimalist conception of the human person, in which autonomy is the only guiding principle (Rendtorff and Kemp 2000, 18–31; Ebbesen 2010, 103). The main disagreements between these two theories relate to 1) Rendtorff and Kemp’s claim that specific ethical principles are needed for Europe, and 2) Rendtorff and Kemp’s charge that Beauchamp and Childress’s theory focuses narrowly on the principle of respect for autonomy (Rendtorff and Kemp 2000, 18–31). According to Rendtorff and Kemp, the principle of respect for autonomy is not sufficient to protect the human person in ethical and legal affairs, because some people might be rendered not autonomous. Principles of dignity, integrity, and vulnerability should therefore supplement respect for autonomy to protect the human person (Rendtorff and Kemp 2000, 18–31; Rendtorff 2002; Ebbesen 2010, 103–104).

It is not clear whether the ethical principles proposed by Rendtorff and Kemp are indeed principles (Beauchamp 2019, 10; Ebbesen 2010, 105). An ethical principle contains an obligation formulated as “you ought to …”. Rendtorff and Kemp’s ethical principles do not include obligations (Ebbesen 2010, 105). Beauchamp writes: “If these are principles, it is unclear to me what makes them principles rather than virtues, conditions, or properties of persons” (Beauchamp 2019, 10). Beauchamp also maintains that autonomy, dignity, integrity, and vulnerability “are not unique for the European context and that, if properly formulated, they are universal values suitable in all countries” (Beauchamp 2019, 10). Beauchamp and Childress defend universality in the common morality and accept ethical pluralism in particular moralities. For instance, in European bioethics (particular morality), the principles included in the common morality might be specified and balanced differently than in American bioethics (particular morality) (Beauchamp 2019, 4).

Regarding Rendtorff and Kemp’s criticism that Beauchamp and Childress’s (2019) theory focuses narrowly on respect for autonomy and the claim by Druedahl, Lebret, and Minssen (2020) that Beauchamp and Childress’s account ignores the principle of solidarity, Beauchamp writes:

This truly basic principle [respect for autonomy]^6^ has been misrepresented in some of the bioethics literature as a principle of individualism, sometimes uncannily characterized as an “American individualism” that emphasizes a liberal political philosophy of individual rights, while neglecting solidarity, social responsibility, social justice, health policy priorities, and the like. But this principle has nothing to do with either individualism or American values. Given the substantial emphasis throughout the book on both beneficence and justice as basic principles, this objection seems to me to pay no attention to what Childress and I have written on the subject. (Beauchamp 2019, 6)

## Method

### A Phenomenological-Hermeneutical Method

We developed a research method specifically to disclose ethical perspectives within biomedical practice (Ebbesen and Pedersen 2007b). This method draws on Lindseth and Norberg (2004) and Pedersen (1999) and is based upon the phenomenological tradition founded by the philosopher Edmund Husserl. According to Husserl, taking a phenomenological approach is characterized by describing phenomena as accurately and completely as possible without explaining, analysing, or making judgements about them (accomplishing epoché), and arriving in this way at the essence of a phenomenon. This is made possible by setting aside common sense and pre-existing scientific knowledge about the phenomenon (Kvale 1983; Ebbesen and Pedersen 2007b). Lindseth and Norberg (2004) take ethically good phenomena as an example: “When the interviewees experience actions, attitudes, relations or other human matters as ethically good or bad, we want to understand this good as the essential meaning of ethically good phenomena (or the essential meaning missing in ethically bad phenomena).” One way of illuminating the essential meaning of phenomena is to tell stories that disclose our experiences about these phenomena (i.e., to narrate from lived experience), to write these stories down, and to interpret them (Lindseth and Norberg 2004; Ebbesen and Pedersen 2007b). Lindseth and Norberg (2004) argue that we have to produce texts to thoroughly examine the meaning structure of phenomena as part of our life world. In the present study, these texts or narratives were produced by conducting narrative interviews.

### Sample

Eight respondents were recruited by purposive sampling regarding their daily work and anonymized (Higginbottom, Pillay, and Boadu 2013). They all held central stakeholder positions, for example, patient, physician, drug industry representative, and politician (table 1). All respondents were acquainted with and had experience in antibody therapy specifically in relation to the treatment of MS. With such a small sample of respondents, this choice served to exclude variability in opinions arising from experience derived from different therapies or diseases. Of course, this leaves open whether other choices in these categories would have changed the results. However, the main aim of this research was to establish whether Beauchamp and Childress’s (2019) theory could structure an analysis of the respondents’ contributions. In our judgement, the benefits in selecting respondents related to a single disease clearly outweighed the concern that such a focus would compromise the validity of insights regarding our aim. Following these principles, several different perspectives on the principles for prioritizing new expensive biological therapies in the Danish healthcare system were illuminated (Bladt et al. 2020). Research projects based on interviews do not have to be reported to the Regional Committee on Health Research Ethics (National Committee on Health Research Ethics 2022).

**Table 1.** Respondents and background information.

**Respondent**	**Background information**
Patient	A woman suffering from MS, which has progressed to secondary MS.
Treating Physician	A physician treating MS patients in the clinic.
MS Association Representative	A representative from the patient organization for Danish MS patients, the Danish Multiple Sclerosis Society.
Physician involved in Drug Regulation	A physician involved in drug regulation in a Danish region, employed at a university and a university hospital.
DMC Representative	A representative from the DMC who prepares recommendations on whether new treatments should be available as standard treatments in Danish hospitals.
Researcher	A researcher employed at a university who studies antibody therapies and develops antibody drugs.
Drug Industry Representative	A representative from the Danish Association of the Pharmaceutical Industry.
Politician	A politician particularly involved in the health care system.

### Semi-Structured Interviews

We used semi-structured interviews to disclose the meaning of lived experience by inspiring the respondents to narrate. Specifically, a semi-structured interview with each respondent was conducted lasting 40–62 minutes (Bladt et al. 2020). The interviews were conducted between November 2017 and January 2018. The interviewer’s questions were open-ended and thematic and formulated before the interview was held. The beginning of the interview was characterized by taking a phenomenological approach, in which the respondent narrated as openly and freely as possible and the interviewer neither analysed nor judged what was said. To encourage further narration, the interviewer asked open-ended questions such as “and what happened next?” Later, the interview was characterized by taking a hermeneutic^7^ approach, in which the interviewer encouraged the respondent to reflect on and interpret what was narrated, for instance by asking “why?” and “how?” (Lindseth and Norberg 2004; Ebbesen and Pedersen 2007b).

### Data Analysis

The research method used for the data analysis was a phenomenological-hermeneutical method for interpreting interview texts that draws on philosopher Paul Ricoeur’s theory of interpretation (Lindseth and Norberg 2004; Ebbesen and Pedersen 2007b; Ricoeur 1976). All interviews were recorded and transcribed verbatim. To enter the hermeneutical circle, the data analysis was divided into three steps: 1) naive reading, 2) structural analysis, and 3) critical interpretation.

#### 1) Naïve reading

The naive reading was an initial reading, in which the text was read several times with a phenomenological attitude to let the text speak to the interpreter on its own. Based on the naive reading, a naive understanding of the text as a whole was formulated. Subsequently, the naive understanding would be either validated or invalidated by the structural analysis (Lindseth and Norberg 2004; Ebbesen and Pedersen 2007b).

#### 2) Structural analysis

Ricoeur stated that to understand a text is to follow its movement from what it says to what it talks about (Ricoeur 1976). In our structural analysis, this movement was followed by reading the whole text and then dividing it into units of meaning (what the text says). These units of meaning were then compared with the naive understanding. Next, the units of meaning were sorted and condensed, and units of significance were formulated (what the text talks about). Lastly, units of significance were condensed yet more to sub-themes and themes (what the text talks about), which were formulated as condensed descriptions and abstract concepts that identify an essential meaning of lived experience. In the structural analysis, the interpreter took a phenomenological approach, that is, in order to view the text as objectively as possible, the units of meaning were decontextualized from the text as a whole and considered individually (Table 2). The thematic approach was judged against the naive understanding to see whether the themes validated or invalidated the naive understanding. If invalidated, steps 1) and 2) were repeated (Lindseth and Norberg 2004; Ebbesen and Pedersen 2007b).

#### 3) Critical interpretation

Gadamer argued that the hermeneutic mode of interpreting meaning is not presuppositionless (Gadamer 2003), that is, texts are not interpreted independent of the interpreter’s tradition of understanding. In research, however, the interpreter must try to make presuppositions and foreknowledge explicit (Kvale 1983). The foreknowledge of the current study included Beauchamp and Childress’s ethical principle of justice (2019). Lindseth and Norberg (2004) state that chosen literature can help to revise, widen, and deepen understanding of an interview text by illuminating the text and, the other way around, the interview text can illuminate the literature (Lindseth and Norberg 2004; Ebbesen and Pedersen 2007b). In this way, we used Beauchamp and Childress’s ethical principle of justice (2019) as an open framework to structure the data analysis.

**Table 2.** **The structural analysis**. Example of the process of how units of meaning evolved into units of significance, sub-themes, and themes for the data collected, i.e., the movement from what was said to what was talked about.

**Respondent**	**Units of meaning (what is said)**	**Units of significance (what is talked about)**	**Themes and sub-themes (what is talked about)**
*The DMC Representative*	“… Equality is an essential ethical principle; hence, equal access to health care. Regardless of where you live in the country, you should be offered equal access to treatments, and uniform treatments should be offered across the country. I hope that it might be possible to offer equal access to treatments across the Regions of Denmark using the guidelines from the Danish Medicines Council”.	Equal access to health care is an essential ethical principle. Uniform treatments and equal access to treatments across the Regions of Denmark might be possible using the guidelines from DMC.	**Allocation within the target health care budget** *Egalitarianism* • Equal access to health care. • Homogeneous treatments and equal access to treatments across the Regions of Denmark.

## Results

### Material Principles of Distributive Justice

All theories of justice include the formal principle of justice that equals should be treated equally, and unequals should be treated unequally. This principle is formal, since it neither offers criteria to decide whether individuals are considered equals nor instructs in which respects equals should be treated equally (Beauchamp and Childress 2019, 268). In contrast, material principles of justice “identify morally relevant properties that persons must possess to qualify for particular distributions” (Beauchamp and Childress 2019, 269). In the present empirical study, at least three theories of justice, each expressing a general material principle of distributive justice, emerged during the data analysis: egalitarianism, utilitarianism, and libertarianism. According to Beauchamp and Childress, the material principle of distributive justice for egalitarianism is: “To each person an equal measure of liberty and equal access to the goods in life that every rational person values” (Beauchamp and Childress 2019, 271); for utilitarianism, it is: “To each person according to rules and actions that maximize social utility” (Beauchamp and Childress 2019, 271); while for libertarianism, it is: “To each person a maximum of liberty and property resulting from the exercise of liberty rights and participation in fair free-market exchanges” (Beauchamp and Childress 2019, 271). These material principles of distributive justice are essential in healthcare prioritization (Beauchamp and Childress 2019, 270–271), and all three principles emerged in the interviews during the data analysis.

The present study investigated the process of prioritizing access to expensive biological treatments in Denmark. The respondents generally focused on their perception of a fair allocation of expensive medicine to patients and fair medicine prices, but they also reflected upon abstract topics related to distributive justice, such as their perception of fair partitioning of the total national social budget and fair allocation within the target healthcare budget. In short, the four themes related to distributive justice that emerged during the data analysis were: 1) partitioning the total national social budget, 2) allocation within the target healthcare budget, 3) allocation of scarce treatments for patients (Beauchamp and Childress 2019, 300–301), and 4) the capitalist market economy and medicine prices. The data analysis below presents these four themes related to distributive justice.

### Theme 1: Partitioning the Total National Social Budget

Governments establish total national social budgets containing allocations for social goods such as healthcare, education, culture, and defence. Since resources are not limitless, expenses for healthcare compete with expenses for other social goods (Beauchamp and Childress 2019, 300). The respondents in the present study agreed that expenditure for medicine in Danish hospitals could not be considered isolated from partitioning the total national social budget. The *Researcher* stated that socio-economic implications of expensive medicine have to be considered but added that patients might have another opinion: “… I think we focus too little on socio-economic considerations in that discussion. And I say this, because I am not sick. If I was sick, I would have a different opinion” (Researcher).

From the perspective of the national social budget, the *Patient* found it fair that major groups of patients suffering from common diseases are prioritized and allocated more resources than minor groups of patients suffering from rare diseases.

Since resources are not limitless, the *Politician* argued that prioritization is essential, and medicine should be given appropriately. The *Politician* also specifically said that the number of chronically ill patients will increase in Denmark in the coming years in an aging population, so that expenses for healthcare will increase correspondingly.

### Theme 2: Allocation Within the Target Healthcare Budget

When the government has allocated money for segments such as healthcare, education, and so on, it must then allocate resources within each segment. In the segment of healthcare, politicians decide which diseases should be given the highest priority in terms of resources allocated (Beauchamp and Childress 2019, 301).

Policymakers will examine various diseases in terms of their communicability, frequency, cost, associated pain and suffering, and impact on length of life and quality of life, among other factors. Under some circumstances, for example, it might be justified, as a matter of priority ranking, to concentrate less on fatal diseases, such as some forms of cancer, and more on widespread disabling diseases, such as arthritis. (Beauchamp and Childress 2019, 301)

This is in line with the statement by the *Researcher*, who expressed the utilitarian view that more resources should be allocated to treatment and research into chronic diseases than to diseases with a low expected survival rate. The *Researcher* argued that cancer-related research receives a priority ranking regarding external funding compared to research into rheumatoid arthritis, which is not a fatal disease (table 3).

**Table 3.** **Allocation within the target health care budget (Theme 2)**. Theories of justice as expressed by the respondents (in italics)

**Respondent**	**Themes related to distributive justice**
Patient	**Allocation within the target health care budget** *Egalitarianism* • Unfair: patients suffering from common diseases receive a high priority ranking.
Treating Physician	**Allocation within the target health care budget** *Egalitarianism* • Regardless of price, patients should have access to the best evidence-based treatments.
MS Association Representative	**Allocation within the target health care budget** *Egalitarianism* • Patients suffering from rare diseases should have access to medicine, care, and support.
Physician involved in Drug Regulation	**Allocation within the target health care budget** *Egalitarianism* • Cancer patients and cancer departments receive high priority rankings. • Pulmonary medicine and psychiatry departments receive low priority rankings. • Inequalities in allocation to specific disease areas are problematic.
Researcher	**Allocation within the target health care budget** *Utilitarianism and egalitarianism* • Fair to allocate more resources to treat chronic diseases rather than diseases with low expected survival rates. • Fair to give best treatment available for the largest possible number of patients, paid by taxes.
Drug Industry Representative	**Allocation within the target health care budget** *Utilitarianism* • Health economics. • Cost-benefit analyses, QALYs. • As many benefits for the money available.
Politician	**Allocation within the target health care budget** *Egalitarianism* • Danish health care system should not exclude treatments that are not cost-effective. • Free and equal access to health care.

#### Cancer Patients Receive High Priority Ranking Versus Equal Access

*The Patient* expressed two different opinions regarding prioritization depending on the perspective from which prioritization was considered. From the perspective of theme 1 (partitioning the total national social budget), the* Patient* found it fair that major groups of patients suffering from common diseases are prioritized and allocated more resources than minor groups of patients suffering from rare diseases. From the perspective of theme 2 (allocation within the target healthcare budget), the *Patient* agreed that cancer patients receive the highest priority ranking regarding allocation of resources in the Danish healthcare system. However, the *Patient* maintained the egalitarian view by emphasizing that from the patient’s perspective, it is not fair that minor groups of patients suffering from rare diseases receive low priority ranking regarding medical examinations and treatments compared to the major group of cancer patients. The *Patient* stated that patients suffering from rare diseases should be given higher priority than they currently have. The *Politician* also maintained the egalitarian view by emphasizing that politicians should ensure patients suffering from rare diseases are prioritized regarding the allocation of resources for research and implementation.

The *Patient* said that there is a lack of human resources in neurological departments in Danish hospitals. The *MS Association Representative* agreed and added that the disease course in patients suffering from MS entails that medical treatment is not possible in some cases. Often, these patients are left without contact with neurologists. The *MS Association Representative* expressed the MS Association’s egalitarian view promoting access for the minor group of MS patients to medicine, care, support, and so on. The *Physician Involved in Drug Regulation* agreed that patients suffering from cancer and cancer-related research receive a high priority ranking regarding resource allocation in the Danish healthcare system and expressed the egalitarian view by emphasizing that such inequalities are problematic. The *DMC Representative* also adhered to the egalitarian material principle of distributive justice: equal access to healthcare is an essential ethical principle, and uniform treatments and equal access to treatments across the Regions of Denmark might be possible using the guidelines from the DMC (text box 1, table 2). The *Researcher* agreed and expressed the egalitarian view by stating that, compared to the American healthcare system (which approximates a libertarian commitment), the Danish healthcare system is fair. The Danish system ensures the best treatment for the largest possible number of patients paid by taxes. The *Researcher* was willing to pay more in taxes to avoid reductions in healthcare financed by the government:

Generally, I think that the Danish healthcare system is the right way to go compared to the American system. I think it is good that we, as a society, ensure that as many patients as possible can get the best treatment. I am willing to pay more in tax to avoid reductions in healthcare financed by the government. Self-financed healthcare entails that there will be groups in society that simply cannot afford it. (Researcher)

The *Treating Physician* also maintained the egalitarian view by stating that patients should have access to the best evidence-based treatments regardless of medicine prices. In contrast, the *Pharmaceutical Industry Representative* supported a utilitarian view by saying that it is essential to get as much benefit as possible from the money available in healthcare. In healthcare economics, cost-benefit analysis is commonly used to compare cost-benefit ratios in different areas of healthcare by using QALYs. However, the *Pharmaceutical Industry Representative* said that the Danish government (which approximates an egalitarian commitment) does not want to use the method of cost-benefit analysis by using QALYs, because this entails a value placed on human life:

… that is the whole way of thinking also with this health economic approach to it, it is of course … utilitarianism, as you point out, that we … it is about getting as much benefit as possible …. (Pharmaceutical Industry Representative)

According to the *Politician*, prioritization in the Danish healthcare system is based on health professional assessment combined with financial assessment. The *Politician* stood up for the egalitarian view by strongly opposing basing prioritization in healthcare solely on cost-benefit analyses and in this way excluding treatments that are not cost-effective (table 3):

… there is no way to make it cheaper. There are ways to discuss whether you can finance it differently, and there are financial ways in which you can slice some off and say, it was a pity they could not afford it. That is how you keep costs down by slicing some off. We do not do that in Denmark. So, the expenditure on health, i.e., real growth, must necessarily increase in the coming years. (Politician)

### Theme 3: Allocation of Scarce Treatments for Patients

Beauchamp and Childress write: “Because health needs and desires are virtually limitless, every healthcare system faces some form of scarcity, and not everyone in need of a particular form of healthcare can gain adequate access to it” (Beauchamp and Childress 2019, 301), so politicians must set priorities. Setting priorities in healthcare includes allocating medical resources to specific classes of patients (Beauchamp and Childress 2019, 301). In Denmark, the DMC decides which patient groups should have free and equal access to every standard medicine.

#### Priority Setting Influenced by Size of Patient Group, Alternative Treatment Options, and Severity of Disease

The *Patient* expressed the egalitarian view by stating that patients suffering from rare and not self-inflicted diseases should receive a high priority ranking regarding access to medicine, even though the expense for medicine per patient suffering from rare diseases is relatively high. The *Politician* also held the egalitarian view. He emphasized that the DMC prepares assessments according to principles stating that patients suffering from rare diseases should be given as high priority as patients suffering from common diseases such as cancer. The *MS Association Representative* argued the egalitarian view by stressing that in assessments of whether new expensive medicine should be recommended as standard treatment, consideration should be given to whether the patient target group has alternative treatment options. If not, the new medicine should receive high priority despite its price. The *Politician* agreed, adhering to the egalitarian principle of distributive justice. It is fair to pay a high price for medicine for a group of patients that previously did not have treatment options:

… if it is effective enough, and it really makes a difference. For instance, that you can help someone that you could not help before, and you can help them in a way that makes a difference. Well, then we are willing to pay a high price … there must be scientific evidence that it has a real good effect. (Politician)

The *DMC Representative* stated that the assessments by the Council adhere to the principle of severity, noting that serious diseases receive high priority ranking (table 4).

**Table 4.** **Allocation of scarce treatments for patients (Theme 3)**. Theories of justice as expressed by the respondents (in italics).

**Respondent**	**Themes related to distributive justice**
Patient	**Allocation of scarce treatments for patients** *Egalitarianism* • Patients should have equal access to expensive medicine. • Patients suffering from rare diseases should receive a high priority ranking regarding access to expensive medicine. • DMC, cost-effectiveness analyses should include total costs including pension, care, etc.
Treating Physician	**Allocation of scarce treatments for patients** *Egalitarianism* • Patients should have equal access to the best evidence-based medicine irrespective of price.
MS Association Representative	**Allocation of scarce treatments for patients** *Egalitarianism* • Patients should have equal access to the best medicine available at a fair price. • DMC, cost-effectiveness analyses should include total costs including pension, care, etc. • Patient groups without alternative treatment options should have prioritized access to expensive medicine. • Problematic to use cost-benefit analyses in health care decision-making. • Problematic to place a value on human life; difficult to quantify the quality of life.
Physician involved in Drug Regulation	**Allocation of scarce treatments for patients** *Egalitarianism* • Patients should have equal access to best-evidence-based medicine irrespective of price. • Danish health care system should offer the best treatment available. • Authorities do not reject effective medicine because it is expensive, and resources are allocated for medicine in hospitals.
DMC Representative	**Allocation of scarce treatments for patients** *Utilitarianism and egalitarianism* • DMC, cost-effectiveness analyses: documentation of effect of new medicine. • DMC’s assessments adhere to severity: serious diseases receive high priority ranking. • Negotiation of fair medicine prices.
Researcher	**Allocation of scarce treatments for patients** Does not express a specific theory of justice.
Drug Industry Representative	**Allocating scarce treatments for patients** *Utilitarianism* • Health economics. • Cost-benefit analyses, QALYs.
Politician	**Allocation of scarce treatments for patients** *Egalitarianism* • Health care system, free and equal access paid by taxes. • Health care decision-making, health professional assessment combined with financial assessment. • DMC’s assessments adhere to 7 principles, not QALYs. • DMC assessments: patients suffering from rare diseases, serious diseases, or who do not have alternative treatment options, should have prioritized access to expensive medicine. • DMC cost-effectiveness analyses should include total costs including pension, care, etc. • New expensive medicine, only marginally better than the standard treatment, should not be implemented.

#### Access to the Best Evidence-Based Medicine

The *DMC Representative* maintained the utilitarian view by emphasizing that the Council prepares cost-effectiveness and cost-benefit analyses of new medicines to be recommended (or not) as standard treatment in Danish hospitals. There are methodological requirements for documentation of the effect of medicines in the assessment process. According to the *DMC Representative*, if the Council’s guidelines are rightly implemented in Danish hospitals, there is room for assessment by the individual physician. This agrees with the statement by the *Politician* that in particular individual cases, health professional assessments can be made that other medication than the standard therapy should be given. However, the *Treating Physician* strongly disagreed that these guidelines leave room to deviate from the standard treatment, stating that the guidelines restrict, rather than just guide, physicians. Physicians are forced to follow the guidelines because failure to adhere to them has financial consequences for the hospital department:

Guideline means that it guides you, without being obligatory. But it is a slightly different situation here, because if we have a guideline, they expect that you really should follow the guideline, and this is mainly for economic reasons. For example, in the MS field, when they introduced the two latest oral treatments, it was teriflunomide, which is Aubagio, and dimethyl fumarate, which is Tecfidera. Then the guidelines suggested that in Denmark about 70 per cent of the patients, who are newly diagnosed and start the treatment should be treated with Aubagio. And this was partly based on economic reasons, and our hospital here enforced this very much for economic reasons. Because Aubagio is cheaper than Tecfidera, so we were really forced to use Aubagio … This immediately limits your choice and your precision medicine. And then it is not a guideline, it is literally not a guideline … if you must follow it. (Treating Physician)

The *Treating Physician* and the *Physician Involved in Drug Regulation* maintained the egalitarian view that patients should have equal access to the best evidence-based medicine irrespective of the price. The *Physician Involved in Drug Regulation* added that patients should be confident that the healthcare system offers the best treatment available:

I am a medical doctor; I do not consider economics. Maybe it is wrong, but I don’t think it is my task to consider that. I would be happy if I was free to give patients the optimal treatment … we are already controlled … Because obviously, my aim is that I would like to be free … to treat the patients according to the best evidence and according to the patient’s needs. (Treating Physician)

The *MS Association Representative* also maintained the egalitarian view that patients should have equal access to the best medicine available and added that fair medicine prices should be negotiated. The *DMC Representative* emphasized that the issue for the Council is not whether patients should be treated or not treated. The point is instead to negotiate favourable medicine prices. This agrees with the *Physician Involved in Drug Regulation*, who stressed that the authorities do not reject effective medicine as standard treatment because it is too expensive. A budget is set for medicine consumption in Danish hospitals. If this budget is exceeded, more resources are allocated, that is, precisely the resources needed for medicine are allocated. However, according to the *Politician*, expensive medicine which is only marginally more effective than the standard therapy is not implemented. This agrees with the statement by the *Researcher* that, previously, patients had access to the best treatment available regardless of price (which approximates to an egalitarian commitment), whereas nowadays the effect of the medicine is evaluated using its cost (which approximates to a utilitarian commitment) (table 4).

#### Cost-Effectiveness and Cost-Benefit Analyses Combined with a More Holistic Perspective

The *MS Association Representative* and the *Patient* believed that the DMC should take a holistic perspective when deciding whether new medicine should be accepted as standard treatment in Danish hospitals. Cost-effectiveness and cost-benefit analyses should compare new medicine to the standard therapy and include a more holistic consideration at the societal level:

We should consider what the expenditures would be, if the medicine was not available, not so much what it would cost the individual hospital or the individual region, but more broadly what the socio-economic costs would be, if an individual did not receive the medicine. In relation to the fact that the person in question might be able to work, there might be lost hours at work, there might be more sick days, there might be all sorts of expenses for aids, which the person in question would not need either … you have to consider more broadly what you get for the money. (MS Association Representative)

The *Politician* agreed that broader perspectives should be included in cost-effectiveness and cost-benefit analyses, contribution to the labour market, and so on. The *MS Association Representative* thought it essential to include patient perspectives and needs and, most importantly, the life quality of the individual patient when deciding whether new medicine should be implemented as standard treatment in Danish hospitals but argued that it is difficult to quantify the quality of life, so it is problematic to use QALYs to value human life. The *Drug Industry Representative* emphasized that health economists and patients have different perspectives. Health economists maintain a utilitarian approach focusing on cost-benefit analyses and QALYs, while patients emphasize that these analyses should be supplemented with reflections regarding life quality for the individual patient. According to the *Politician*, the DMC was established without using QALYs and that the use of QALYs meant healthcare prioritization would be based on financial assessments only. The *Politician* emphasized that healthcare prioritization should be based on health professional assessment combined with financial review (table 4).

### Theme 4: The Capitalist Market Economy and Medicine Prices

In the present study, the respondents generally acknowledged that prices for medicine are high because of high costs for pharmaceutical companies for research, development, trials, etc., but they also believed that medicine prices tend to increase rapidly and that it is fair that Danish authorities should regulate medicine prices to a reasonable level. The *DMC Representative* specifically pointed out that while expenditures for medicine are high and increasing, departments in Danish hospitals lack resources for nurses and care in general, so it is important to both prioritize and negotiate favourable medicine prices. According to the *Pharmaceutical Industry Representative*, Denmark is unique in having price negotiations and agreements that set fixed upper prices for medicine:

Considered worldwide, it is unique that we have these price agreements in Denmark. This is our contribution to the expenses not running wild. Prices are rising, they may well rise … for instance, we see prices for hospital medicines are rising. With the price cap, however, we ensure that the pharmaceutical industry does not raise prices, so that only the volumes of medicine increase because a larger number of patients are treated. (Pharmaceutical Industry Representative)

However, *the Physician Involved in Drug Regulation* emphasized that when authorities push down medicine prices, there is a risk that pharmaceutical companies will not want to enter the Danish market. For instance, this is the case in Norway, where some medicines are unavailable for Norwegian patients:

The Medicines Council has just said no to Spinraza. And then we can ask, well, is the company interested in doing something regarding the price? This is the first time someone is trying to push down prices in this country. If enough countries say no, then it will probably change … However, there is a … we have seen in Norway, where they have said no for a long time. There are drugs that do not enter Norway … that the Norwegians simply do not get. These drugs do not enter the market in Norway, because they say no. So, obviously, this is a risk. (Physician Involved in Drug Regulation).

According to the *Treating Physician*, negotiating medicine prices is complicated, because multiple interests are involved: pharmaceutical companies demand profit, and society wants medicine available for patients. According to the *Politician*, when the patent period of a drug expires, it means free-market competition. When the patent period for an original drug has expired, biosimilars enter the market, leading to meagre prices for medicine. The *Politician* stated that this shows that the pharmaceutical industry is lucrative. According to the *Researcher*, since biosimilars undergo reduced clinical trials, the biosimilar price is lower than the original product price. Reduced clinical trials enrol fewer subjects and aim only to test the toxicity, not the drug’s effect, so the price of a biosimilar is approximately half of the original product price. However, the *Treating Physician* cautioned against prescribing biosimilars, because one does not know whether the biosimilar is similar to the original product because of the reduced clinical trials.

## Validity and Relevance of the Study

### Validity

To address the validity of a qualitative study, it is important to present a clear description of methods for the collection of data, data analysis, and interpretation (Mays and Pope 2000). In the current study, section "Method" presents a transparent explanation of the research process from data collection to analysis and interpretation. Regarding data analysis and interpretation, table 2 provides an account of the process of how units of meaning evolved into units of significance, sub-themes, and themes for the data collected (Mays and Pope 2000). As pointed out earlier, the hermeneutic mode of interpreting meaning is not presuppositionless (Gadamer 2003). To address the validity of the current study, it is important to make the presuppositions and foreknowledge of the study explicit (Kvale 1983). In particular, the foreknowledge of the present study was Beauchamp and Childress’s ethical principle of justice (2019). However, to show sensitivity to the ways that presuppositions and foreknowledge might shape the data collected (Mays and Pope 2000), we let the interview text illuminate the literature and vice versa. In this way, we avoided forcing the literature perspective onto the interview text (Lindseth and Norberg 2004; Ebbesen and Pedersen 2007b). Furthermore, this study explored the narratives, including the opinions of the respondents. For instance, the interviewer did not correct the respondents during the interviews, because that might have shaped the data.

On the other hand, the current study was also limited by the presuppositions and foreknowledge described above. For instance, the concepts of libertarianism, egalitarianism, and utilitarianism were defined as described by Beauchamp and Childress (2019). Certainly, there are different views presented in the literature, but the arguments for selecting Beauchamp and Childress’s theory (2019) as an open framework to structure the data analysis are thoroughly described in section "Theory".

### Relevance

Research can be relevant when it supplements already acquired knowledge or increases confidence in this knowledge (Mays and Pope 2000). The current study explored Beauchamp and Childress’s theory of principles (2019), which is accepted as an important philosophical contribution and used worldwide for ethical assessments. The ambition of the current study was to illuminate whether real-life opinions with regard to prioritization of expensive medicine in Denmark could be analysed using Beauchamp and Childress’s theory (2019) as an open framework to structure the data analysis. The limited number of respondents were used to this end. Indeed, it seems that at least some of Beauchamp and Childress’s (2019) principles can be applied here (Bladt et al. 2020). The value of our work is in encouraging the use of these principles in larger investigations. Our aim was not to generalize beyond the setting in which the findings were generated (Mays and Pope 2000). Informants were recruited by purposive sampling regarding their daily work (Higginbottom, Pillay, and Boadu 2013). They all occupied different central stakeholder positions, and they were familiar with and had experience in antibody therapy. In this way, we explored diverse perspectives on the principles for prioritizing new expensive biological therapies in a Danish context (Bladt et al. 2020).

## Discussion

### Material Principles of Distributive Justice Expressed by the Respondents

Seven out of eight respondents maintained an egalitarian view regarding allocation within Denmark’s target healthcare budget. These respondents emphasized that 1) patients should have free and equal access to the best treatment available, 2) healthcare should be paid for by taxes, and 3) inequalities in allocation to specific disease areas are problematic. In contrast, the last respondent, the *Drug Industry Representative*, maintained the utilitarian view that allocation within the target healthcare budget should be based on cost-benefit analyses and QALYs (table 3). The same pattern was seen regarding the allocation of scarce treatments for patients, where seven out of eight respondents expressed the egalitarian view that 1) patients should have equal access to expensive medicine, and 2) patients suffering from rare diseases, serious diseases, or who have no alternative treatment options should receive high priority ranking regarding access to expensive medicine. Again, the eighth respondent, the *Drug Industry Representative*, expressed the utilitarian view that access to expensive medicine should be based on cost-benefit analyses and QALYs (table 4). So, in this study, the respondents across stakeholder positions generally expressed the egalitarian view that patients should have free and equal access to the best treatments available, including costly medicine. Moreover, patients suffering from rare diseases, severe diseases, or those without other treatment options should be given high priority regarding access to expensive medicine.

### Utilitarian Cost-Effectiveness, and Cost-Benefit Analyses in Healthcare Prioritization

Next, we examined the use of utilitarian cost-effectiveness and cost-benefit analyses in healthcare prioritization. As indicated above, the *Drug Industry Representative* expressed the utilitarian view that allocation within the target healthcare budget and that access to expensive medicine should be based on cost-benefit analyses and QALYs (tables 2 and 3). Four respondents specifically argued that access to expensive medicine should be based on cost-effectiveness and cost-benefit analyses but that these analyses should also consider the severity of the disease, alternative treatment options, frequency of disease cases, and total costs such as expenses for care, pension, and so on (table 4).

According to Beauchamp and Childress, utilitarian cost-effectiveness and cost-benefit analyses fail to attend adequately to important justice concerns such as the needs of the disabled and the worst-off patients regarding disease severity. Nevertheless, they argue that cost-effectiveness and cost-benefit analyses bring important information for public policies if constrained by the right to a decent minimum of healthcare and the fair opportunity rule (Beauchamp and Childress 2019, 255–256, 293). Some of these considerations are reflected in the DMC principles and important factors for prioritizing expensive medicine in Danish hospitals, according to which cost-effective analyses must be constrained by concerns regarding geographical equality, access to treatment, severity, and caution (text box 1). However, our empirical study indicated that patients suffering from cancer and cancer-related research receive a high priority ranking, whereas patients suffering from rare diseases receive low priority ranking in the Danish healthcare system.

This confirms the DCE criticism that the Danish healthcare system unfairly prioritizes patients suffering from cancer compared to other diseases. As they say, it is unfair to apply the type of disease as a criterion for prioritization (Danish Council on Ethics 2018, 3–17). Both the DCE and the respondents in the present study emphasize the importance of equal access for patients regardless of which disease they have. Furthermore, the DCE requests principles for prioritization in the Danish healthcare system (Danish Council on Ethics 2018, 3). Lastly, therefore, we explored whether Beauchamp and Childress’s (2019) principle of justice is helpful as a conceptual framework for reflecting on the prioritization of new expensive biological therapies in the Danish healthcare system.

### The Principle of Justice as a Conceptual Framework

In Beauchamp and Childress’s (2019) theory, the egalitarian concerns of equal access to treatment and disease severity are more or less incorporated in the fair opportunity rule and the universal right to a decent minimum of healthcare. This corresponds to the first tier of the healthcare model proposed by Beauchamp and Childress, which is financed by the government and covers “basic and catastrophic health needs” (Beauchamp and Childress 2019, 293). The Danish healthcare system corresponds to the first tier of this healthcare model but covers more than just primary and acute healthcare. Indeed, according to the Danish Health Act, the Danish healthcare system must promote the overall health of the population in Denmark and prevent and treat illness, pain, and disability in the individual patient. Furthermore, the Danish healthcare system is required to meet the need for easy and equal access to healthcare (Danish Health Act 2019). Since 2017, however, the DMC has prepared recommendations on whether new treatments should be available as standard treatments in Danish hospitals based on cost-effectiveness and cost-benefit analyses with the constraints of geographical equality, access to treatment, severity, and caution (DMC 2022). Since 2017, therefore, the use of utilitarian cost-effectiveness and cost-benefit analyses with egalitarian constraints has been incorporated to determine what a decent minimum of healthcare entails in the Danish healthcare system. However, in line with the Danish Health Act (2019), the respondents in the present study requested free and equal access to government-funded primary and acute healthcare and expensive medicine.

## Conclusion and Future Perspectives

The respondents in this study generally maintained the egalitarian view that patients should have equal access to the best treatments available, including expensive medicine financed by the government. Moreover, they felt that patients suffering from rare diseases, severe diseases, and those with no other treatment alternatives should receive high priority regarding access to costly medicine. The egalitarian concerns of equal access to treatment and disease severity are roughly reflected in the fair opportunity rule and the universal right to a decent minimum of healthcare in Beauchamp and Childress’s (2019) model (Tier 1), but this government-financed tier only covers primary and acute healthcare. Our empirical study indicates that, to be helpful in a Danish context, Tier 1 of Beauchamp and Childress’s (2019) model should include equal access to government-funded primary and acute healthcare, including costly medicine, and other scarce treatments. Furthermore, Tier 2 in the model proposed by Beauchamp and Childress (2019), should give access to healthcare at private hospitals at personal expense. If modified in these respects, Beauchamp and Childress’s principle of justice (2019) might be helpful as a conceptual framework for reflections on the prioritization of expensive biological therapies in the Danish healthcare system.

This study included eight respondents who were recruited by purposive sampling regarding their daily work, which was related to antibody therapy. All the respondents occupied central stakeholder positions, for instance, patient, physician, drug industry representative, and politician. Future perspectives could be to recruit more respondents, so that a group of respondents for each central stakeholder position is formed. Such a study could be interpreted in the light of the broader literature and might for instance explore whether different kinds of egalitarianism are at stake.

## Footnotes

1 Since resources of societies are limited, priorities need to be set for healthcare. Beauchamp and Childress therefore consider the right to a decent minimum of health care an attractive goal “… and, realistically, probably the only goal that can be achieved in most societies seeking to implement a right to health care” (Beauchamp and Childress 2019, 292–293).

2 In this article, our purpose is to explore principles for just prioritization of expensive biological therapies in the Danish healthcare system. This aim includes exploring the use of data gained from analytic techniques in general in the light of distributive justice (Beauchamp and Childress 2019, 253). These analytic techniques are briefly exemplified by cost-effective and cost-benefit analyses. Strictly speaking, however, cost-effective analyses and cost-benefit analyses are two different approaches (Ubel 2000; Lippert-Rasmussen and Lauridsen 2010).

3 Cost-benefit analysis (CBA) (Beauchamp and Childress 2019, 253).

4 In this article, MS patients are considered a minor patient group suffering from a rare disease. In Denmark approximately 17,200 patients, or approx. 3:1000, suffer from MS (Danish Sclerosis Association 2022), which would not qualify MS as a rare disease according to the European Union definition with a threshold at 1:2000 (European Commission 2021). However, in the public debate, the concept of rare disease seems to follow a more relational understanding, where it is noted that the prevalence of MS is dwarfed by cancer, type II diabetes, and heart diseases. This is also evident from our interviews and discussed in the present paper with this understanding.

5 The issue of what equal access requires in the context of rare diseases is thoroughly discussed in the literature (Albertsen 2022; Juth 2017; Juth et al. 2021; Magalhaes 2022; Sandman and Gustavsson 2017).

6 Inserted by the authors.

7 Hermeneutics is the study of the interpretation of texts. The aim of hermeneutics is to gain a valid and common understanding of a text (Kvale 1983).
